# Letter to the editor Re: “Cultivating a thriving environment for women in cardiology through leadership and inclusion”

**DOI:** 10.1016/j.ahjo.2025.100583

**Published:** 2025-07-24

**Authors:** Shreeya Mehta

**Affiliations:** King's College London, United Kingdom of Great Britain and Northern Ireland

Dear Editor,

As a final year female medical student with a strong interest in cardiology, I read with great curiosity the article by Bullock-Palmer et al., “*Cultivating a thriving environment for women in cardiology through leadership and inclusion*” [1]*.* I commend the authors for their comprehensive and thought-provoking article shedding urgent light on a persistent crisis in the field of cardiology: the striking underrepresentation of women in cardiology despite decades of advocacy and incremental progress.

As a society with increasing cardiovascular disease, we cannot ignore the striking numbers that only 13 % of US attending cardiologists and less than 10 % of interventional and electrophysiology specialists are women [2]—numbers that have barely budged over the past decade.

What makes this gender disparity even more poignant is the mounting evidence that women physicians provide better patient centred care [3], yet the structural barriers for women to pursue this specialty remain formidable.

Without visible female cardiology role models, the hostility and prejudices are likely to remain. More female cardiology role models are vital for the new generation of doctors and could be a pivotal part in attracting more female medical students to the field of cardiology. It is telling—and unacceptable—that only 5 % of U.S. division chiefs in cardiology are women, a statistic that has not changed since 1992 [4].

Despite the ACC and AHA having launched exemplary initiatives, such as mentorship networks and leadership workshops, it is imperative that we move from awareness to measurable accountability. Institutions and societies must:

1. Set concrete representation benchmarks for women in leadership, with clear reporting.
2. Mandate enforcement and education around protections such as Title VII, the Family and Medical Leave Act, and the Pregnant Workers Fairness Act.
3. Expand sponsorship pipelines, ensuring women are actively recommended for guideline authorship, trial leadership, and high-visibility roles.
4. Champion cultural change, so that the next generation views cardiology not as an exclusionary specialty but as a welcoming, innovative community.

This article is a powerful call to action, but its message must catalyse structural change. If we fail to cultivate environments where women can not only enter but *thrive* in cardiology, we fail our patients and our profession.

## CRediT authorship contribution statement

**Shreeya Mehta:** Conceptualization, Writing – original draft, Writing – review & editing.

## Declaration of competing interest

The authors declare that they have no known competing financial interests or personal relationships that could have appeared to influence the work reported in this paper.
